# Incidence of and Risk Factors for Tuberculosis (TB) in Gastric Cancer Patients in an Area Endemic for TB: A Nationwide Population-based Matched Cohort Study: Erratum

**DOI:** 10.1097/01.md.0000496597.44876.e4

**Published:** 2016-09-09

In the article “Incidence of and Risk Factors for Tuberculosis (TB) in Gastric Cancer Patients in an Area Endemic for TB: A Nationwide Population-based Matched Cohort Study”,^1^ which appeared in Volume 94, Issue 47 of *Medicine*, data in the abstract was originally presented incorrectly. The article has since been corrected online.
